# Reviewing the Analytical Methodologies to Determine the Occurrence of Citrinin and Its Major Metabolite, Dihydrocitrinone, in Human Biological Fluids

**DOI:** 10.3390/molecules25122906

**Published:** 2020-06-24

**Authors:** Liliana Silva, André Pereira, Sofia Duarte, Angelina Pena, Celeste Lino

**Affiliations:** 1 LAQV, REQUIMTE, Laboratory of Bromatology and Pharmacognosy, Faculty of Pharmacy, University of Coimbra, Polo III, Azinhaga de Stª Comba, 3000-548 Coimbra, Portugal; amptpereira@gmail.com (A.P.); s.cancela.duarte@gmail.com (S.D.); apena@ci.uc.pt (A.P.); cmlino@ci.uc.pt (C.L.); 2 Vasco da Gama Research Centre—Department of Veterinary Sceinces, Escola Universitária Vasco da Gama, Av. José R. Sousa Fernandes, Campus Universitário—Bloco B, 3020-210 Coimbra, Portugal

**Keywords:** citrinin, dihydrocitrinone, biomonitoring, urine, blood, plasma, analytical methodologies

## Abstract

Until now, the available data regarding citrinin (CIT) levels in food and the consumption of contaminated foods are insufficient to allow a reliable estimate of intake. Therefore, biomonitoring configuring analysis of parent compound and/or metabolites in biological fluids, such as urine or blood, is being increasingly applied in the assessment of human exposure to CIT and its metabolite, dihydrocitrinone (DH-CIT). Most studies report urinary levels lower for the parent compound when compared with DH-CIT. A high variability either in the mean levels or in the inter-individual ratios of CIT/DH-CIT between the reported studies has been found. Levels of DH-CIT in urine were reported as being comprised between three to seventeen times higher than the parent mycotoxin. In order to comply with this objective, sensitive analytical methodologies for determining biomarkers of exposure are required. Recent development of powerful analytical techniques, namely liquid chromatography coupled to mass spectrometry (LC-MS/MS) and ultra-high-performance liquid chromatography (UHPLC-MS/MS) have facilitated biomonitoring studies, mainly in urine samples. In the present work, evidence on human exposure to CIT through its occurrence and its metabolite, in biological fluids, urine and blood/plasma, in different countries, is reviewed. The analytical methodologies usually employed to evaluate trace quantities of these two molecules, are also presented. In this sense, relevant data on sampling (size and pre-treatment), extraction, cleanup and detection and quantification techniques and respective chromatographic conditions, as well as the analytical performance, are evidenced.

## 1. Introduction

Mycotoxins, secondary toxic metabolites, are produced by some fungal species, such as *Aspergillus*, *Penicillium*, *Fusarium* and *Alternaria*, in both crops and processed food commodities under favorable conditions namely of moisture, temperature and water activity [1,2].

Among the more than 400 mycotoxins already identified and reported, those that attracted notable attention for the toxic effects and high prevalence in the agro-food commodities are aflatoxins (AFs), ochratoxin A (OTA), trichothecenes (deoxynivalenol (DON) and nivalenol (NIV)), fumonisins (FBs), zearalenone (ZEN), patulin (PAT) [1,2,3] and citrinin (CIT) [2,3].

Citrinin (CIT) was first discovered in 1931 due to its antibiotic properties but it was never used because of its mammalian toxicity [4]. This polyketide mycotoxin is produced by fungi belonging to the genera *Penicillium*, *Aspergillus* and *Monascus* [5]. It was first isolated from *Penicillium citrinum* before World War II. Afterward, it was identified in several *Penicillium* (e.g., *P. citrinum*, *P. expansum*, *P. radicicola*, *P. verrucosum*) and *Aspergillus* (e.g., *Aspergillus terreus* and *Aspergillus niveus*) species, as well as certain strains of *Penicillium camemberti* and *Aspergillus oryzae*. CIT has also recently been isolated from *Monascus ruber* and *Monascus purpureus* [6,7]. Dihydrocitrinone (DH-CIT) was isolated in 1962 from a mutant strain of *Aspergillus terreus* that did not produce CIT, being later demonstrated as a regular metabolite in both *Penicillium citrinum* and *Aspergillus carneus* [8].

Cereals, such as rice, wheat, oats, rye, corn and barley, were described as the foods containing higher CIT levels [6]. However, it has also been found in black olives [9,10], fermented meat products [11], cheese [12] and apples [13].

Exposure to CIT is of toxicological concern and its toxicity has been thoroughly investigated. CIT disturbs the kidney function in several species, namely in the renal tubules [14]. CIT induced micronuclei in human-derived liver cells (HepG2) at levels equal to or greater than 10 μM and decreased in a dose-dependent way the percentage of binucleated cells [15].

In Japan, CIT has been related with yellow rice disease. It was further implicated as causative contributor in swine nephropathy. Although recognized as a nephrotoxin in all tested animal species, its acute toxicity is variable across the different species [6,16]. CIT is an acknowledged nephrotoxin that affects monogastric species like pigs and dogs [17]. However, on humans the effects of this mycotoxin are not yet fully known. Synergistically with ochratoxin A (OTA), CIT can decrease the synthesis of RNA in the kidneys of murine models [6].

Studies regarding CIT toxicity recognized the kidney as the main target organ following repeated dose exposure in animals. Thus, CIT resembles OTA, although clearly featuring lower nephrotoxic potency [14]. Co-occurrence of these two nephrotoxins in different commodities of the animal and human food chain raised concerns about the potential risks to human and animal health [18]. CIT and OTA are associated with the etiology of porcine nephropathy and of Balkan endemic nephropathy (BEN) in humans [19,20]. However, due to limited evidence for carcinogenicity in experimental animals CIT was classified by IARC in group 3 [7,14].

Following the biotransformation of CIT, the main metabolite formed appearing in the human urine is dihydrocitrinone (DH-CIT) [21,22]. The toxicity of DH-CIT was investigated, and the studies revealed that its cytotoxic potency was clearly lower compared with CIT as well as its genotoxic potential [23]. The metabolism of CIT in DH-CIT can thus be considered as a detoxification reaction.

**Figure 1:**
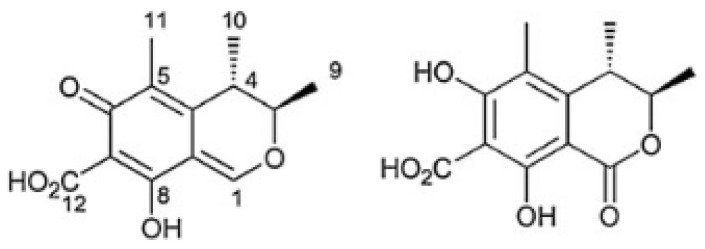
Citrinin (CIT, on the **left**) and dihydrocitrinone (DH-CIT, on the **right**) [22,26].

Chemically, CIT is a quinone featuring two intramolecular hydrogen bonds [24]. It is identified as (3*R*,4*S*)-8-Hydroxy-3,4,5-trimethyl-6-oxo-4,6-dihydro-3H-isochromene-7-carboxylic acid, the chemical formula C_13_H_14_O_5_ (Figure 1) and CAS Number 518-75-2. The molecular weight of CIT is 250.25 g/mol [5,16]. DH-CIT, also known as HO-CIT, is (3*R*,4*S*)-6,8-Dihydroxy-3,4,5-trimethyl-1-oxo-3,4-dihydro-1*H*-isochromene-7-carboxilic acid and the chemical formula C_13_H_14_O_6_ (Figure 1) [18]. Citrinin is described has solid lemon-yellow needles, with color changing according to the pH in solution. It is lemon-yellow at pH 4.6 whereas at pH 9.9 it is cherry red. The melting point of CIT is 178.5 °C. In cold water it is practically insoluble whereas in hot water it is moderately soluble. It is soluble in the majority of polar organic solvents, including methanol, ethanol and acetonitrile, as well as in aqueous sodium hydroxide, sodium carbonate and sodium acetate [24,25].

Biomarkers of exposure either in urine and/or plasma are considered a useful tool in the evaluation of the internal mycotoxin exposure in humans [15]. Biomarkers are particularly useful whenever food analysis data is limited or inadequate, as in the case of CIT. Thus biomonitoring, as the analysis of human biological fluids for suitable biomarkers of exposure reflecting the dietary intake, permit a valuable understanding on human exposure to this mycotoxin [15,18].

This paper presents a review of different studies on human exposure to CIT worldwide, through the occurrence of the mycotoxin and of its metabolite, DH-CIT, in human urine and plasma. In this sense, relevant data on sampling, such as size and pre-treatment, extraction and cleanup strategies are reported. The use of adequate and sensitive analytical methodologies for detection and quantification and respective chromatographic conditions, as well as the analytical performance achieved, for both molecules, is evidenced. The vast majority of methods applied so far in the evaluation of CIT biomarkers rely on LC-MS/MS analysis. However, some authors also applied liquid chromatography–fluorescence detector (LC-FD), as an alternative given that, due to its planar structure, CIT has natural fluorescence [24]. Many different sample preparation strategies aiming the selective enrichment of the analytes have been described in literature are widely employed nowadays, namely liquid-liquid extraction (LLE) and solid phase extraction (SPE). Direct ‘dilute-and-shoot’ (DaS) approaches have been tried but its efficiency can be compromised when low concentrations of the analyte are present. Furthermore, the co-eluted matrix components can affect ionization of the target analytes.

A comprehensive review has been conducted covering the last decade of published references in this topic. Several database platforms such as PubMed, Science Direct and Google Scholar were used for the search. The keywords used individually or in combination in the search list, were citrinin, occurrence, biological fluids and analytical methodologies. Due to the lack of data on the presence of CIT in breast milk [27], this biological fluid was not included in this review.

## 2. Occurrence in Biological Fluids

A suitable risk assessment of CIT is hampered by the very incomplete knowledge on human dietary exposure to this mycotoxin [14]. Whenever food contamination data are limited or unavailable, biomonitoring constitutes a valuable and effective strategy in the assessment of human CIT exposure. However, it relies on sensitive analytical methods and information on toxicokinetics [28].

Internal exposure assessed through biomonitoring analysis of target chemicals, metabolites or reaction products in urine or plasma can relate exposures to health outcomes. Regular methods are suitable for biologically persistent chemicals. However, the effective evaluation of short-lived chemicals depends on a continuous exposure or knowledge of the timing of exposures in the individual. In particular, urinary excretion mainly represents recent mycotoxin intake, reflecting short-term exposure with more day-to-day variation [29], whereas measurements in plasma/serum are more likely to represent long-term exposure [30].

### 2.1. Urine

Urine is oftentimes the matrix of choice for the ease of collection. Nevertheless, the different fluid intakes result in different urine excretion. Such limitation can be mitigated through normalization for the creatinine concentration in the samples. For exposure assessment, collection of 24-h urine is recommended. Studies on the stability of many different target analytes showed that these were stable up to 12 h at 25 °C after collection [31]. However, it is recommended a conservation at 2–4 °C to prevent fermentation that could affect the sample components [30].

As mentioned before, data on contamination of food with CIT is scarce which preclude the evaluation of dietary exposure. Despite availability of data resulting from urinary biomonitoring performed in different countries, the lack of information on the toxicokinetics of CIT hinders the estimation of daily intake [26].

After rapid absorption, CIT is distributed namely to the liver and kidney. According to a recent study on human toxicokinetics, 40% of CIT underwent urinary excretion and thus absorption was equal to or higher than 40% [32]. CIT toxicokinetics in humans following oral intake were further reported [27]. After ingestion, CIT is converted into the main metabolite DH-CIT, being both excreted in the urine. In 24 h the cumulative urinary excretion ranged between 32.9% and 70.8% (median 40.2%) of the sum of CIT and DH-CIT (‘total CIT’). In urine, the median half-life of CIT and DH-CIT was reported as 6.7 h and 8.9 h, respectively. CIT was excreted in urine after 20 to 22.5 h of administration. The greatest excretion pattern of CIT and DH-CIT after administration in humans was set at 22.5 h [26]. Nevertheless, data concerning the CIT metabolism and excretion rate resulted from a study enrolling two single participants, with highly variable results [26]. The two volunteers, both health females, identified as individual A and B, ingested CIT twice (spaced out some weeks) at doses lower than the TDI. The half-lives of the biomarkers in urine were estimated as 5.5 h and 6.3 h (in the case of CIT; mean: 6.7 h) and 7.5 h and 9.2 h (in the case of DH-CIT; mean: 8.9 h), for individual A and B, respectively [26].

Conversely to the limited literature on the toxicokinetics and metabolism of CIT in humans, in rats DH-CIT was isolated and effectively identified as the predominant urinary metabolite of CIT already in 1983 [31]. In the urine of rats CIT was not detected [32].

Table 1 shows CIT and DH-CIT data, known so far, in human biological fluids in different countries. Overall, most studies report urinary levels lower for the parent compound when compared with the major metabolite (DH-CIT). The high variability in either the mean levels or the inter-individual ratios of CIT/DH-CIT is also noticeable [33]. Levels of DH-CIT in urine were reported as being three [5], five [18], ten, thirteen and seventeen times [34] greater than the parent mycotoxin. Although the enzymes that catalyze such detoxification reaction are not yet known, it is recognized that this conversion can be variable individuals. Therefore, it is recommended that biomonitoring studies should include analysis of both CIT and DH-CIT and interpret results in light of individual and combined (sum of both analytes) levels [24,30].

In human urine, the un-metabolized mycotoxin was detected at low levels (ranging from 2 to 5 ng/mL), demonstrating the possibility of urinary excretion, although at low levels. Such feature hinders the detection of CIT in food and biological samples in routine analysis.

In Belgium, CIT was detected in a single sample among the 40 samples analyzed, with a contamination level of 6.8 ng/mL (value un-corrected) [31]. Another study evaluated the urine of a small cohort comprising 4 adults (German) and 6 infants (Turkish) [22]. In the ten urines analyzed, CIT was detected (above the limit of quantification (LOQ) in 8 samples whereas DH–CIT was detected in the ten samples but with values above the LOQ in half of the samples. According to such results, urinary excretion occurred mainly in the form of the un-metabolized mycotoxin and to a lesser extent in the form of DH-CIT.

A similar study performed in fifty German health adults, of which 27 women and 23 men reported a widespread and variable exposure to CIT [5]. The un-metabolized mycotoxin was detected in 82% of the samples, varying between 0.02 (limit of detection (LOD) and 0.08 ng/mL, with a mean level of 0.03 ng/mL. DH-CIT was found in 84% of the samples, ranging between 0.05 (LOD) and 0.51 ng/mL, with a mean level of 0.10 ng/mL. The adjustment to creatinine content resulted in three times higher level of the metabolite DH-CIT (60.9 ng/g) compared to the parent compound (20.2 ng/g), thus supporting its usefulness as a CIT exposure biomarker [5]. Further studies demonstrated the widespread occurrence of DH-CIT in urine, reflecting an extensive conversion of CIT to DH-CIT and thus supporting an effective human metabolism of CIT [23]. Also, in Germany, a study evaluated exposure to CIT of female and male workers in three grain mills in North Rhine Westphalia, through collection of spot urine. A third cohort corresponded to male controls [39]. Although both mycotoxin and metabolite were detected in all samples analyzed, DH-CIT showed average levels three times higher (0.045 vs. 0.14 µg/g creatinine). Furthermore, the median levels of the metabolite (110 ng/g creatinine) were equally superior to those of CIT (40 ng/g creatinine), in the three groups enrolled. This study thus reinforced the value of this less toxic metabolite in the human exposure assessment of CIT [39].

In Belgium, first morning urine samples of 32 volunteers were analyzed for CIT and DH-CIT. It was observed a widespread contamination, with 90% of the urine samples contaminated though at low levels (pg/mL). CIT was detected in 59% of the samples at levels up to 117 pg/mL whereas DH-CIT, considered a detoxification product and a major hepatic metabolite of CIT, was detected in 66% of the samples at levels up to 208.5 pg/mL [23,44]. A further study in the same country, showed that 72% of adult (*n* = 239) and 59% of children (*n* = 155) analyzed urine samples contained CIT at low mean levels (<73.3 pg/mg creatinine). The DH-CIT mean levels were higher than the parent compound in both adults (752.0 vs. 56.7 pg/mL; 13 times higher) and children (550.7 and 31.4 pg/mL; 17 times higher). Conversely, the contamination frequency was lower when compared with the parent compound, in both adults (12% vs. 59%) and children (6% vs. 72%) [34].

In Portugal, the National Food, Nutrition and Physical Activity Survey of the Portuguese General Population (2015–2016), conducted through analysis of urine paired samples (24 h urine and first-morning urine) of 94 individuals revealed a low exposure to CIT. Indeed, the mycotoxin was only detected in 2% of both samples, from two participant women, with one surpassing the established TDI (HQ = 1.04) [38]. In a further study in Portugal, the mycotoxin and the major metabolite were evaluated in the urine of controls (*n* = 19) and workers (*n* = 21) of a fresh bread dough company [37]. CIT was found in one control sample above the LOQ (5%) and in 10 samples (53%) < LOQ. DH-CIT was found in 2 samples between the LOD and LOQ and 17 < LOD. Among the employees, 3 urine samples contained DH-CIT levels between LOD and LOQ and 18 < LOD. Occupational exposure was also evaluated in 25 employees of swine farms, through analysis of urine samples. Eight per cent and 12% were contaminated with CIT and DH-CIT, respectively. The control group (*n* = 19) showed a lower number of quantifiable results (<LOQ) [45].

Recently, CIT and DH-CIT exposure was evaluated in fifty Czech patients diagnosed with malignant renal tumors [36]. CIT and DH-CIT were detected in 91% (ranging from nd to 87 ng/L) and 100% (ranging from 6 to 160 ng/L of the urine samples, respectively. Urine samples (*n* = 1096) from Swedish adolescents showed that 1.5% of the population was contaminated with DH-CIT [46].

A study performed in Bangladesh showed a widespread contamination along with five times higher levels of DH-CIT in urine when compared with CIT. Despite the individual variability observed in the ratios of urinary CIT/DH-CIT [1.3–6.6], the metabolite proved to be a valuable biomarker of exposure to CIT. Among the 69 urine samples evaluated, CIT was detected in 94% (up to 1.22 µg/L) and the metabolite DH-CIT in 71% (up to 7.47 µg/L). The results also showed that contamination levels were higher among the rural population in comparison with the urban population. Although uncertain, such difference probably related with diverse eating habits and/or occupational exposure [18].

Also, in Bangladesh, exposure to CIT among pregnant women living in both rural and suburban are of the Savar region (District of Dhaka) was evaluated. It was observed a widespread contamination in the 54 spot urine samples analyzed, with 87% contaminated with CIT at levels varying between 0.02 and 6.93 ng/mL. In all the analyzed samples, the levels of the DH-CIT metabolite were 1.5 times higher when compared with CIT According to the residency, in the rural area 84 and 84% and in the suburban area, 91 and 86% of the samples were contaminated with CIT and DH-CIT, respectively (Table 1). Contamination levels of both compounds were two times higher among the rural population when compared with the suburban population, thus supporting the results of the previously mentioned study [41]. As a result, the estimated daily intake for the rural population was higher than for the suburban population. For CIT, the preliminary TDI is established at 0.2 μg/kg body weight per day based on no concern for nephrotoxicity in humans [14]. The authors reported that 9% (*n* = 3) of the pregnant women from the rural area (*n* = 32) surpassed the proposed TDI. The highest EDI calculated among the pregnant women living in the rural area was 1.09 μg/kg bw/day, which is thus 5 times higher that the proposed TDI. No significant association was observed between exposure biomarkers in urine and the food commodities consumed. Nonetheless, total CIT levels were greatly higher in the pregnant women consuming more rice daily [41].

A biomonitoring survey for DH-CIT in human urine samples from Bangladesh, Germany and Haiti was reported. The average concentration (2.75 ng/mL) among the Bangladeshi participants was higher among the 76% contaminated urine samples, when compared with the German and Haitian participants (0.12 and 0.49 ng/mL) [40]. In a previous study conducted in the German city of Munster, the authors found a single sample containing DH-CIT from the 101 urine samples collected from healthy volunteers [47].

In Bangladesh, a biomonitoring follow-up study was carried out to examine potential regional and seasonal determinants of CIT exposure [33]. Detection of the parent compound revealed noticeable variations. The contamination levels were higher for DH-CIT than for CIT, in both summer (0.42 ± 0.98 vs. 0.10 ± 0.17 ng/mL) and winter (0.59 ± 0.98 vs. 3.18 ± 8.49 ng/mL) collected samples. The rural population presented higher total CIT biomarker levels when compared with the urban population, whether in summer or winter season. However, the average concentration of CIT and DH-CIT was significantly (*p* < 0.05 and *p* < 0.01) higher among the rural cohort only during summer. In wintertime, only the average concentration of the metabolite was the significant different (*p* < 0.001) between rural and urban populations [33]. The EDI of CIT was calculated as 0.043 ± 0.099 μg/kg b.w./day in the summer and 0.304 ± 0.776 μg/kg b.w./day in the wintertime. It was found that 10% (in summer) and 24% (in winter) of the enrolled participants surpassed the preliminary TDI established by EFSA (0.2 μg/kg b.w./day). The worst-case scenario calculated among the participants corresponded to an EDI of 4.66 μg/kg b.w./day. As in a previously cited study [40], apart from for a positive trend observed between CIT biomarkers in urine and increased rice consumption, no significant correlations were observed [33].

According to a study performed in urines from volunteers in northern Nigeria, the positivity and levels of both CIT (incidence: 66%; mean: 5.96 ng/mL; max: 241 ng/mL) and DH-CIT (incidence: 58%; mean: 2.39 ng/mL; max: 17 ng/mL), were considerably higher in comparison with previous reported studies [42,48].

CIT analysis in urine from Tunisian population showed an average CIT concentration higher in colorectal cancer (CRC) patients than in controls, for mean levels either uncorrected or corrected for creatinine (ng/mg), 0.45 ± 0.24 vs 0.44 ± 0.21 and 0.95 ± 1.43 vs 0.53 ± 0.48, respectively [15]. In Wuhan, China, 60 urine samples from control volunteers (*n* = 30) and hepatocellular carcinoma patients (HCC) (*n* = 30) were evaluated. The concentrations were lower than the LOQ (0.3 µg/L) [49].

### 2.2. Blood/Plasma

Through the assessment of exposure biomarkers of CIT in the blood/plasma levels, information on internal circulation is made available, however the steady-state concentration has to be taken into account [43]. In plasma the median half-life for CIT is estimated as 9.4 h. A provisional daily CIT intake was assessed based on both excretion of ‘total CIT’ in urine and reported urine biomarker information in different population groups. It was found that whereas the European surveyed population groups featured an exposure clearly below the TDI, in Bangladesh one biomonitoring study demonstrated that the TDI was surpassed for one population group [26].The levels found in plasma (0.90 ng/mL) were higher than in urine (0.78 ng/mL) [26].

Biomonitoring data of CIT in human blood, plasma or serum is still scarce, as shown in Table 1. In Germany, CIT was detected in all the plasma samples provided by four men and four women, with levels varying between from 0.10 to 0.25 ng/mL. Despite its small-scale, this biomonitoring study supported a widespread exposure of Germany adults to CIT [22].

In Czech Republic, a high incidence of CIT (98%) was also found in plasma samples from kidney tumor patients, with levels up to 182 ng/L (mean: 0.061 ± 0.035 ng/mL) [36]. In Tunisia, mean levels of CIT in plasma of both controls and patients with CRC was higher than the ones reported in previous studies. It was also noticeable that the levels between controls and patients with CRC did not differ [15]. The results from a survey in 60 plasma samples collected from HCC patients and control volunteers, in China (Wuhan), showed that only one sample from patients with HCC was contaminated, with a content of 0.63 µg/L, a value very close to the LOQ, 0.44 µg/L [49].

Human exposure to CIT was also evaluated through plasma biomarker in Bangladesh [43]. The biomonitoring study analyzed CIT and DH-CIT in plasma from University students collected in two seasons: summer (June 2013; *n* = 64) and winter (March 2014; *n* = 40). CIT was found in higher frequency (90% vs. 85%) and maximum concentration levels (2.70 vs. 1.44 ng/mL) in comparison with DH-CIT. It was also observed that the mean levels, of either CIT or DH-CIT were higher in winter (0.47 vs. 0.40 ng/mL) than in summer (0.25 vs. 0.37 ng/mL). Currently no information of the toxicokinetics of CIT is available and so it is not possible to estimate the dietary intake based on plasma biomarker levels [14,33]. It is also important to notice that according to a pilot study performed in a single volunteer, following the ingestion of a small dose of CIT, a cumulative excretion of roughly 36% (12% corresponding to CIT and 24% to DH-CIT) was observed through analysis of 24 h urine. Such value has been applied before, among a Bangladeshi cohort, in the estimation of probable daily CIT intake values based on the urine levels found. In this way it was estimated that 24% of the enrolled population surpassed the TDI as proposed by EFSA (2012; 0.2 μg/kg bw/day) [14,33]. This is of concern, when taking into account that among the University student enrolled in the study cited before [43] it was also found a frequent co-occurrence to an additional mycotoxin OTA with acknowledged nephrotoxic effects [43].

## 3. Analytical Methodologies

Sampling, such as size and pre-treatment, extraction, clean-up as well as adequate and sensitive analytical methodologies for detection and quantification are key steps for the analysis of CIT and its metabolite in human biological fluids (urine and blood). Table 2 shows the sample size and its pre-treatment, extraction and clean-up procedures, detection and quantification methods, as well as the respective chromatographic conditions and the limits of detection (LODs) and quantification (LOQs) obtained for each methodology used so far for determination of these compounds in human biological fluids.

### 3.1. Urine

#### 3.1.1. Sample Pre-Treatment

The sample size varies between 500 µL [42] and 20 mL [22,34,50]. Prior to the extraction process, several researchers used centrifugation to remove solid residues (Table 2). The centrifugal forces or spin speeds used are variable. Some of them used 4000× *g* for 10 min [31,34,50], 5600× *g*/3 min, followed by incubation with 500 µL PBS (pH 7.4) containing β-glucuronidase for 16 h at 37 °C [42], 14,000× *g* during 10 min [40] or 16,800× *g* for 5 min [34]. Others employed 3940 rfc [22] or 10,000 rpm for 5 min [50]. A dilution with 5 mL of 1 mM acetic acid in water followed by a mixture for 15 min was performed by some researchers [5,18], while others opted by a mixture of ACN/H_2_O/FAc in different proportions [15].

#### 3.1.2. Extraction and Clean-Up Procedures

A liquid-liquid extraction (LLE) with ethyl acetate acidified with formic acid, completed with SAX-SPE clean-up has proven to be a suitable pre-analytical set up [31] (Table 2). Furthermore, it is an alternative to the expensive and broadly employed immunoaffinity columns (IACs). When the obtained results with clean up by SAX and Oasis HLB SPE columns were compared, it was observed that the LODs were 3–9 times lower with SAX cartridges. Such difference could be ascribed to a better ability of the SAX columns to remove the matrix interferences, thus improving sample clean-up [31].

Blaszkewicz et al. [22], after centrifugation and urine acidification with an aqueous solution of acetic acid, used for SPE clean-up, C18 cartridges for HPLC-FD analysis and CitriTest™ IAC columns before liquid chromatography coupled to mass spectrometry (LC-MS/MS) analysis. Various authors used these procedures to assess CIT and DH-CIT in human urine in different countries [5,18,33,39,41].

Aliquots of 10 mL were centrifuged (16,800× *g*, 5 min) and passed through a RC syringe filter. After, an IAC cleanup with CitriTest™ columns was used. Elution was achieved with 2 mL acetone acidified with HCl (0.1 mol/L), followed by evaporation. A 10 μL aliquot was injected in a ultra-high-performance liquid chromatography (UHPLC-MS/MS) instrument, with a gradient common to the direct method [35]. This analytical methodology presented high sensitivity, allowing LODs of 0.001 and 0.010 ng/mL for CIT and DH-CIT, respectively and LOQs of 0.003 and 0.030 ng/mL, for CIT and its metabolite, respectively.

The extraction efficiency and the ability to reduce matrix interference in MS/MS chromatograms were compared between Oasis^®^ HLB and PRiME HLB columns [42]. PRiME HLB columns were chosen since exhibited better extraction efficiency, S/N ratios and a faster processing time. Furthermore, equilibration of the column allowed a faster clean-up. Different eluents were also evaluated namely MeOH (100%), ACN (100%), MeOH/ACN (50%/50%) and in some cases acidified with 1% HAc. Pure ACN resulted in higher extraction efficiency and elution was performed by using three times a volume of 200 µL [42].

A QuEChERS-based procedure previously developed by Vidal et al. [51] was recently applied [38]. In a 50 mL centrifuge tube, 2 mL of urine were diluted in 18 mL of ACN/H_2_O/FAc (52/45/3, *v*/*v*/*v*). The mixture of 4 g of magnesium sulphate and 1 g of sodium chloride was followed by a 30 min vigorous agitation of the tube on a rotary shaker. The mixture was then centrifuged at 4000× *g* during 6 min. Five mL of the resulting organic layer were evaporated to dryness under a gentle N_2_ stream at 40 °C. The obtained residue was reconstituted in 0.5 mL of injection solvent (H2O/MeOH, 85/15, *v*/*v*), filtered through a PVDF membrane with 0.22 μm pore size and analyzed by LC-MS/MS [38]. A similar procedure, with variations in solvent proportions, was also used in order to evaluate CIT levels in colorectal cancer patients in Tunisia [15]. Curiously, the last method showed better sensitivity (Table 2).

Heyndrickx et al. [34,50] used one simplified methodology in which, after a 5 min centrifugation (10,000 rpm), 2 mL of urine was passed through a syringe filter (0.2 μm). Gerding et al. [40,47], after centrifugation, simply diluted the supernatant by a factor of 10 in H_2_O/ACN/FAc before injection in the LC-MS/MS system. This rapid “dilute and shoot” (DaS) technique was also applied to evaluation of DH-CIT in urine samples from Swedish adolescents [46]. A simple DaS approach was also used to analyze urine samples (200 μL) that were mixed with 730 μL of ACN/H_2_O (10/90, *v*/*v*). Afterward, the diluted sample was filtered with a syringe filter (0.22 μm; Nalgene™). Aliquots of 10 μL were analyzed by LC–MS/MS [49].

#### 3.1.3. LC-FD and LC-MS-MS Quantification

As shown in Table 2, detection and quantification by LC-MS/MS became more common. LC-FD may be an alternative given that, due to its planar structure, CIT has natural fluorescence, presenting highest fluorescence at pH 2.5 by its nonionized molecule [24]. Chromatographic conditions are shown in the above mentioned table. Reverse phase is used, being the solid phase constituted by a nonpolar adsorbent, such as a C18 column and by a polar mobile phase. The column temperatures used are variable, usually between 21 °C [22] and 40 °C [35]. Regarding flow rates, when UPLC is used higher flow rates are employed, 800 L/h [33] or 550 L/h [15,38].

When LC-MS/MS or UPLC-MS/MS were used several mobile phase gradients were attempted. Several authors reported the used of water/formic acid (99.7/0.3, *v*/*v*) and methanol/water/formic acid (94.7/5/0.3, *v*/*v*/*v*), including both 5 mM ammonium formate [31,34,50]. The mixture of 1 mM ammonium formate in water and 1 mM ammonium formate in MeOH was chosen by Blaszkewicz et al. and Ali et al. [5,18,22]. A quaternary system of water, methanol, 10% acetic acid in water and 500 mM ammonium acetate in water acidified with 5% acetic acid was also reported [35]. Acetonitrile and water, both acidified either with 0.1% formic acid [40,47] or 0.1% acetic acid [42] were also described. A mixture of water/methanol/acetic acid (94/5/1, *v*/*v*/*v*) and methanol/water/acetic acid (97/2/1, *v*/*v*/*v*), both buffered with 5mM ammonium acetate [38] were applied. When LC-FD was employed the following mobile phases were used: water/methanol/acetic acid (96%) (69.5:30:0.5, *v*/*v*/*v*) and methanol/acetic acid (96%) (99.5:0.5, *v*/*v*) [22].

The great majority of authors use multiple reaction monitoring (MRM) with electron spray ionization (ESI) in negative mode (−) when UPLC [15,35,38,42] or HPLC [5,18,22,36,40,43] is used. More rarely, some researchers use MRM-ESI in positive mode (+) [31,50].

Two distinctive MRM transitions are usually used to safeguard an accurate identification [40]. The precursor ion chosen for CIT vary between 249 (*m*/*z*) [5,22] and 281 *m/z* [35,38]. The product ions, for the first precursor ion, were 204.7 *m*/*z* and 176.7 *m*/*z* [5,15,22] and for the second were 249 and 205 *m*/*z* [35,38].

For DH-CIT, the precursor ion selected by different authors oscillated between 265 *m*/*z* [5,22,35,42] and 267 *m*/*z* [40]. The products ions obtained for the first precursor ion were 221 and 176.7 *m*/*z* [5,22,35]. Šarkanj et al. [42] selected as product ions 221.1 and 246.9 *m*/*z*. For 267 *m*/*z* precursor ion, the obtained products were 203 and 231 *m*/*z* [40].

According the chromatographic conditions, retention times for CIT oscillated between 7.3 [22] and 18.9 min [42] and for DH-CIT varied between 6.8 [22] and 15.9 min [42].

For CIT, the LOD values oscillated between 2.88 [31] and 0.001 ng/mL [34,35,50] and for DH-CIT among 0.1 [40] and 0.003 ng/mL [42]. The LOQ values for CIT were comprised between 5.76 [31] and 0.003 ng/mL [35] and for DH-CIT between 0.2 [15] and 0.01 ng/mL [42].

### 3.2. Blood/Plasma

#### 3.2.1. Sample Pre-Treatment, Extraction and Clean-Up

For blood, a 15 min centrifugation at 3000 rpm (roughly 1620× *g*), after addition of 2.7 mL K_2_EDTA, allowed to obtain 4 mL of plasma. For plasma analysis, a protein precipitation step is required. Blaszkewicz et al. [22] mixed 1 mL of plasma with acetonitrile (1:1, *v*/*v*), followed by 3 min centrifugation at 9300 rcf. One mL of the upper layer was evaporated to dryness under a gentle nitrogen flow at 40 °C. The residue was then dissolved in 300 µL methanol before chromatographic analysis [22].

Similarly, Malir et al. [36] mixed 1 mL of plasma with 1 mL ACN (1/1, *v*/*v*) before a 3 min centrifugation at 9800× *g*. One mL of the obtained upper layer was evaporated to dryness under a gentle N_2_ flow, at 40 °C. The residue was then dissolved in 350 μL of MeOH, shaken in a vortex and centrifuged once more at 9800× *g* during 3 min. The resulting extract was passed through a Teflon syringe filter (0.45 μm) [36].

For plasma separation, Ali et al. [43] also used centrifugation, at 3000 rpm (≅ 2500× *g*) during 15 min. For protein precipitation, 1 mL of plasma sample was mixed with 1 mL ACN (1:1, *v*/*v*). Additional centrifugation at 12,000 rpm during 3 min followed. One mL of the resulting upper layer was evaporated to dryness under a gentle nitrogen flow at 40 °C. The residue was dissolved in 350 μL of MeOH, shaken in a vortex and centrifuged once more at 12,000 rpm during 3 min. The resulting extract was finally passed through a Teflon syringe filter (0.45 μm) [43].

Conversely to the results obtained for urine, the DaS procedure is not the most adequate for blood samples, given the possibility of forming blood clots and contaminate MS system. Therefore, in order to reduce as much as possible the sample preparation procedure, deproteinization with *β*-glucuronidase is possible [49].

Recently, as shown in Table 2, a QuEChERS methodology, similar to that used for urine samples, was also applied to plasma [15].

#### 3.2.2. LC-FD and LC-MS-MS Quantification

For detection and quantification of CIT, LC-FD was used by Blaszkewicz et al. (2013) [22]. However, its presence at low concentrations in complex matrices, became LC-MS/MS the preferred method. Chromatographic conditions match those applied in urine samples and are specified in Table 2. For plasma, some methods exhibited identical LOD and LOQ values for CIT as for its metabolite [22,36,43], as shown in Table 2. One method showed higher sensitivity, being the LOD 0.04 ng/mL and the LOQ 0.09 ng/mL [15]. Conversely, the methodology proposed by Cao et al. [49] presented lower sensitivity, with LOD and LOQ values of 0.18 and 0.44 ng/mL, respectively. It is acknowledged that the reliability of these methods is frequently compromised by matrix effects resulting in ion suppression or enhancement. According to a recent study of matrix effects in multi-residue analysis of mycotoxins in biological specimens a signal enhancement occurred in the case of CIT and suppression in the case of the metabolite DH-CIT [52]. It is therefore highly recommended the use of suitable, preferably isotope-labeled, internal standards for accurate quantification. In the last years, ^13^C-labeled reference standards for regulated mycotoxins become commercially available [53]. Stable isotope labeled internal standards, such as isotope labeled citrinin (^13^C_15_ CIT), were used especially in challenging biological matrices, as urine and plasma [15,26,42,43]. This use has the advantage to correct potential losses of mycotoxin throughout the analysis [15].

## 4. Final Remarks

Studies on the incidence of CIT and its major metabolite CH-CIT in biological fluids reveal a wide dissemination in different continents, such as Europe, Asia and Africa. Regarding urine, the data obtained so far reveal that the highest levels of CIT were found in Nigeria, 5.96 ± 27.43 ng/mL [42], followed by Bangladesh, in winter, in a rural area, 0.66 ± 0.91 ng/mL (1.16 ng/mg creatinine) [33] and in Tunisia, 0.45 ± 0.24 ng/mL (0.95 ng/mg creatinine) in patients with colorectal cancer [15]. Concerning DH-CIT, the highest levels were found in a rural area of Bangladesh, in the winter, 5.95 ± 1.63 ng/mL (7.23 ± 12.2 ng/mg creatinine) [33], followed by 2.75 ± 8.43 ng/mL (3.12 ng/mg crea) in the same area [40] and from Nigeria, with 2.39 ± 3.56 ng/mL [42].

With respect to plasma matrix, DH-CIT was evaluated, so far, in only one study, in Bangladesh, in winter, reporting a mean level of 0.4 ± 0.33 ng/mL [43]. CIT revealed higher plasma levels in Tunisia than in Bangladesh, 0.50 ± 0.19 ng/mL vs. 0.47 ± 0.5ng/mL [15,43].

Nowadays, LC/MS/MS-based methodologies are selected in order to reach the most required low detection limits for CIT and DH-CIT in biological fluids. However, matrix effects can present impairments for these methods. This can be avoided by efficient extraction and clean-up steps, usually consisting of centrifugation followed by solid phase extraction with C18 [22], SAX [31], polymeric [31,42] or IAC cartridges [5,18,22,33,39,41] or through the use of QuEChERS-based procedures [38,51]. The DaS approach is a promising pre-analytical step in urine [47,49] or plasma [49], primarily in the presence of high levels of target analytes. However, it is required the absence of co-elution and interference of matrix components with ionization of the target analytes. Furthermore, the wide spectra of DaS compromises sensitivity [54]. Such effects can be compensated through the use of stable isotope-labeled standard of CIT. So, stable isotope dilution assays (SIDA) can circumvent part of these issues [8] thus facilitating biomonitoring studies [28].

## Figures and Tables

**Table 1:** CIT and DH-CIT occurrence in human biological fluids in different countries.

Biological Fluid	Country	No of Samples	Incidence (%)		Range (ng/mg Creatinine)		Range (ng/mL) Un-Corrected		Mean ± SD (ng/mL) Un-Corrected		Mean ± SD (ng/mg Creatinine)		Median (ng/mL) Un-Corrected		References
			CIT	DH-CIT	CIT	DH-CIT	CIT	DH-CIT	CIT	DH-CIT	CIT	DH-CIT	CIT	DH-CIT	
Urine	Belgium (Gent)	40	2.5	na	nd–4.5	na	nd–6.8	na	na	na	na	na	na	na	[31]
	Belgium	32	59	66	na	na	<LOQ (0.002)–0.117	<LOQ (0.030)–0.2085	0.026	0.035	na	na	na	na	[35]
	Belgium—Children	155	72	6	0.002–0.4157	0.2688–2.029	0.0016–0.3928	0.2594–0.8873	0.0314	550.7	0.0398	0.8102	0.0212	0.4943	[34]
	—Adults	239	59	12	0.0016–1.4943	0.0929–2.4656	0.0022–1.398	0.1431–2.1177	0.0567	752.0	0.0737	0.739	0.0176	0.5603	
	Czech Republic—Kidney tumor patients	50	91	100	na	na	0.002–0.087	0.006–0.160	0.016 ± 0.020	0.048 ± 0.034	0.022 ± 0.021	0.084 ± 0.077	0.008	0.038	[36]
	Portugal—Controls	19	12.03	2.11	na	na	na	na	na	na	na	na	na	na	[37]
	—Workers of one fresh bread dough company	21	6.29	3.14	na	na	na	na	na	na	na	na	na	na	
	Portugal Adults—24 h Urine	94	2	na	na	na	nd–1.20	na	na	na	na	na	0.85	na	[38]
	—First-morning urine	94	2	na	na	na	nd–1.0	na	na	na	na	na	0.75	na	
	Germany	4 M	100	100	na	na	<LOQ–0.07	<LOQ–0.34	na	na	na	na	na	na	[22]
	Germany—Adults	27 F	74	78	nd–0.120	nd–0.480	nd–0.07	nd–0.43	0.03 ± 0.02	0.10 ± 0.10	0.0294 ± 0.0267	0.1035 ± 0.1077	0.02	0.05	[5]
		23 M	91	91	nd–0.1905	nd–0.5484	nd–0.08	nd–0.51	0.04 ± 0.02	0.11 ± 0.11	0.0399 ± 0.0446	0.100 ± 0.1158	0.04	0.07	
		50 total	82	84	nd–0.1905	nd–0.5484	nd–0.08	nd–0.51	0.03 ± 0.02	0.10 ± 0.10	0.0342 ± 0.036	0.102 ± 0.1104	0.03	0.06	
	Germany—Volunteers (controls, IfAD staff)	13	100	100	0.006–0.196	0.006–0.568	0.010–0.178	0.010–0.460	0.050 ± 0.043	0.139 ± 0.131	0.061± 0.055	0.150± 0.148	0.039	0.098	[39]
	—Workers in three grain mills: M	12	100	100	0.006–0.062	0.022–0.720	0.009–0.076	0.042–0.211	0.031 ± 0.019	0.110 ± 0.054	0.030± 0.015	0.140 ± 0.187	0.025	0.111	
	—Workers in three grain mills: F	5	100	100	0.007–0.059	0.032–0.383	0.007–0.056	0.006–0.506	0.028 ± 0.019	0.158 ± 0.198	0.034 ± 0.019	0.142 ± 0.143	0.024	0.008	
	Germany	50	na	28	na	na	na	<LOQ–0.33	na	0.12 ± 0.02	na	0.09	na	0.10	[40]
	Haiti	142	na	14	na	na	na	<LOQ–4.34	na	0.49 ± 0.95	na	0.28	na	0.27	
	Bangladesh	95	na	75	na	na	na	<LOQ–58.82	na	2.75 ± 8.43	na	3.12	na	0.42	
	Bangladesh (Rajshahi district)—Rural area	32	97	91	na	na	nd–1.22	nd–7.47	0.14 ± 0.22	0.97 ± 1.75	na	na	0.08	0.20	[18]
	—Urban area	37	92	54	na	na	nd–0.45	nd–0.36	0.06 ± 0.07	0.08 ± 0.09	na	na	0.03	0.05	
	Bangladesh (Summer)—Rural area (Mongol Para, Puthia)	30	97	93	na	na	nd–1.22	nd–5.39	0.14 ± 0.22	0.78 ± 1.33	0.53 ± 0.80	2.81 ± 6.15	0.08	0.20	[33]
	—Urban area (Rajshahi University region)	32	90	50	na	na	nd–0.45	nd–0.31	0.06 ± 0.08	0.08 ± 0.08	0.20 ± 0.21	0.31 ± 0.27	0.03	0.04	
	—Total samples	62	95	71	na	na	nd–1.22	nd–5.39	0.10 ± 0.17	0.42 ± 0.98	0.36± 0.60	1.52 ± 4.43	0.05	0.10	
	Bangladesh (Winter)—Rural area (Mongol Para, Puthia)	30	93	97	na	na	nd–3.51	nd–46.44	0.66 ± 0.91	5.95 ± 1.63	1.11 ± 1.63	7.23 ± 12.20	0.28	0.65	[33]
	—Urban area (Rajshahi University region)	32	91	97	na	na	nd–5.03	nd–4.64	0.52 ± 1.05	0.60 ± 1.02	0.85 ± 1.90	2.86 ± 1.60	0.18	0.21	
	—Total samples	62	92	97	na	na	nd–5.03	nd–46.44	0.59 ± 0.98	3.18 ± 8.49	0.97 ± 1.76	3.94 ± 9.07	0.21	0.27	
	Bangladesh Pregnant women—Rural area	32	84	84	na	na	na	na	0.42 ± 1.2	0.55 ± 1.04	(ng/g) 0.60 ± 1.21	(ng/g) 0.70 ± 0.70	0.17	0.22	[41]
	—Suburban area	22	91	86	na	na	na	na	0.15 ± 0.13	0.23 ± 0.18	0.39 ± 0.57	0.57 ± 0.69	0.22	0.18	
	—Total samples	54	87	85	na	na	na	na	0.31 ± 0.93	0.42 ± 0.82	0.51 ± 0.99	0.65 ± 0.69	0.13	0.18	
	Nigeria	120	65.8	57.5	na	na	0.015–241.46	0.05–16.89	5.96 ± 27.43	2.39 ± 3.56	na	na	0.84	1.00	[42]
	Turkey	6 I	100	100	na	na	<LOQ–0.2	<LOQ–1.12	na	na	na	na	na	na	[22]
	Tunisia—Controls	50	72	na	<LOQ–5.72	na	<LOQ–0.98	na	0.44 ± 0.21	na	0.53 ± 0.48	na	na	na	[15]
	—Colon rectal cancer	50	76	na	<LOQ–2.94	na	<LOQ–0.96	na	0.45 ± 0.24	na	0.95 ± 1.43	na	na	na	
Plasma	Germany (Dortmund)	4 M	100	na	na	na	0.10–0.25	na	na	na	na	na	na	na	[22]
		4 F	100	na	na	na		na	na	na	na	na	na	na	
	Czech Republic—Kidney tumor patients	50	98	na	na	na	nd–0.182	nd	0.061 ± 0.035	nd	na	na	0.051	na	[36]
	Bangladesh—Midsummer	64	86	84	na	na	nd–1.96	nd–0.93	0.25 ± 0.31	0.37 ± 0.24	na	na	0.18	0.33	[43]
	—Winter	40	97	85	na	na	nd–2.7	nd–1.44	0.47 ± 0.50	0.4 ± 0.33	na	na	0.31	0.3	
	—Total samples	104	90	85	na	na	nd–2.7	nd–1.44	0.34 ± 0.4	0.38 ± 0.27	na	na	0.22	0.31	
	Tunisia—Controls	50	34	na	na		<LOQ–0.84	na	0.50 ± 0.19	na	na	na	na	na	[15]
	—Colon Rectal Cancer	50	38	na	na		<LOQ–0.94	na	0.47 ± 0.2	na	na	na	na	na	

F—female; LOQ—limit of quantification; M—male; na—not available; nd—not detected.

**Table 2:** Analytical methods for determination of CIT and/or DH-CIT in human biological fluids.

Biological Fluids	Sample Size	Sample Pre-Treatment	Extraction	Clean-Up	Detection and Quantification	Chromatographic Conditions	LOD ng/mL	LOQ ng/mL	References
**Urine**	10 mL	Centrifugation at 4000× *g*; 10 min.	**1—LLE:** with 15 mL ethyl acetate/FAc (99/1, *v*/*v*), shaking (30 min). Centrifugation (4000× *g,* 10 min). Evaporation of ethyl acetate phase into a new extraction tube and dried (40 °C). pH adjustment of the aqueous phase (acidified urine) between 6.5 and 7 with **Na_2_CO_3_** (0.4 M). Dilution of urine (1/5, *v*/*v*) in MeOH. Passed through a pre-conditioned SAX SPE cartridge for sample clean-up. **2**—dilution of 10 mL urine with ultrapure water (1/1, *v*/*v*).	**1—SAX SPE:** Wash with 1 mL water. Elution: 5 mL of acidified MeOH (1% FAc). The eluate was combined with the residue obtained after LLE with ethyl acetate. Evaporation of the pooled extract at 40 °C. The residue (from combined fractions) was dissolved in 200µL of injection solvent (H_2_O/MeOH/FAc 61.8/37.9/0.3, *v*/*v*/*v*). Hexane (500 uL) was added and shaken (1 min). The content was brought into a centrifugal filter (Millipore) and centrifuged (14,000× *g*, 15 min). A 150 uL aliquot of the aqueous phase was injected into UPLC-MS/MS. **2—Oasis HLB:** Elution with 10 mL DCM/MeOH (70/30, *v*/*v*) containing **50 mM HCl**. Aspiration of the colored upper phase into a clean test tube containing 5 mL of ethyl acetate/TFA (99/1, *v*/*v*). After shaking the mixture was centrifuged (3000× *g*, 3 min). The ethyl acetate/TFA (99/1, *v*/*v*) phase was carefully aspirated and combined with the DCM/MeOH extract. This combined solution was then evaporated to dryness at 40 °C. The final residue was reconstituted in 200 µL of the injection solvent.	**UPLC–MS/MS** triple quadrupole multiple reaction monitoring mode (MRM) Ionization: ESI+	**Column:** C18 100 mm × 2.1 mm i.d., 3.5 µm, Zorbax SB. **Guard column:** 10 mm × 2.1 mm i.d., 5 µm, Zorbax Eclipse XDB-C8. **Mobile phase: A:** water/FAc (99.7/0.3, *v*/*v*); **B:** MeOH/water/FAc (94.7/5/0.3, *v*/*v*/*v*); both containing 5 mM ammonium formate. **Injection volume:** 20 µL **Source temperature:** 130 °C **Desolvation temperatures:** 350 °C **Desolvation gas flows:** 800 L/h **Cone voltage:** 25 V **Collision energy:** 40 Ev **RT (min):** 9.13 **Precursor ion (*m*/*z*):** 251.50; **Product ions (*m*/*z*):** 90	CIT:2.88	CIT:5.76	[31]
**Urine**	20 mL	Centrifugation at 3940 rcf	5 mL urine mixed with 5 mL of 1 mM acetic acid in water.	**C18:** elution with 3 mL MeOH. Evaporation to dryness at 40 °C. Dissolution of the residue in 500 µL MeOH. Filtration through Teflon syringe filter (0.45 µm) **IAC:** elution with 4 mL of MeOH. Evaporation to dryness (40 °C). Dissolution of the residue in 500 µL MeOH.	**HPLC-FD:** λ exc. 330nm; λ em. 500 nm **LC-MS/MS** Quadrupole MS/MS equipped with an ESI source	**Column:** C18 Nucleodur Sphinx EC 125/3 (3 µm material) at 22 °C. **Mobile phase:** **A:** water/MeOH/HAc (96%) (69.5:30:0.5, *v*/*v*/*v*); **B:** MeOH/HAc (96%) (99.5:0.5, *v*/*v*). Flow rate: 0.4 mL/min **RT(CIT):** 12.5 min **—Column:** Nucleosil^®^ 100-5 C18 HD 125×3 mm, at **21 °C**. **Mobile phase:** **A:** ammonium formate 1 mM in water; **B:** ammonium formate 1 mM in MeOH. Flow rate: 0.3 mL/min. **RT(CIT):** 7.3 min; **RT(DH-CIT):** 6.8 min **Injection volume:** 40 µL ESI–MS/MS was executed by MRM in negative ion mode **Collision energy:** 16 and 20 eV **Transitions:** **CIT:** 249.0→204.7 *m/z* and 249.0→176.7 *m*/*z* **DH-CIT:** 265.0→221.0 *m*/*z* and 265.0→176.7 *m*/*z*	CIT: 0.02 DH-CIT: 0.05	CIT: 0.05 DH-CIT: 0.10	[22]
**Urine**	10 mL	Centrifugation (16,800× *g*, 5 min).	Filtration through a RC syringe filter.	**IAC Citritest:** Elution with 2 mL ACT acidified with 0.1 mol/L HCl. Mixture of the eluent with 100 μL of DMSO (keeper solvent). ACT evaporation at 45 °C. Remaining DMSO solution: centrifugation (16,800× *g*, 10 min).	**UHPLC-MS**	**Column:** Waters Acquity UPLC^®^ HSS T3 2.1 × 100 mm, 1.8 μm, at 40 °C. **Guard-column:** C18, 1.7 μm, 2.1 mm × 5 mm. **Mobile phase:** **A:** water **B:** methanol **C:** 10% acetic acid in water **D:** 500 mM ammonium acetate in water acidified with 5% acetic acid. For the analysis in negative mode, mobile phase C was continuously added at a rate of 1% resulting in a constant 0.1% acid concentration throughout the run. **Injection volume:** 10 μL **Flow rate:** 500 μL/min **Cone voltage:** 60 V **Transitions:** CIT: 281/249/205 *m*/*z* DH-CIT: 265/221/177 *m*/*z*	CIT: 0.001 DH-CIT: 0.010	CIT: 0.003 DH-CIT: 0.030	[35]
**Urine**	20 mL	Centrifugation at 10,000 rpm; 5 min	2 mL: filtration with a syringe filter (0.2 μm). *Filter and shoot*	---	**UHPLC-MS** Triple quadruple MRM mode Positive ESI mode	**Column:** Waters HSS T3 (100 mm × 2.1 mm; 1.8 µm). **Mobile phase:** **A:** water **B:** MeOH both containing 5 mM ammonium formate and 0.05% acetic acid **Injection volume:** 10 µL	CIT: 0.001 DH-CIT: 0.010		[34,50]
**Urine**		Centrifugation at 14,000× *g*; 10 min	100 μL supernatant diluted with 900 μL H_2_O/ACN/FAc (0.94/0.05/0.01, *v*/*v*/*v*). *Dilute and shoot* (DaS)	---	**LC-MS/MS** QTRAP mass Spectrometer ESI negative mode MRM	**Column:** Nucleodur^®^ C18 Pyramid column (3 μm, 2.0 × 150 mm). **Guard column:** C18 EC (2 mm × 4 mm). **Mobile phase:** **A:** ACN (0.1% formic acid) **B:** water (0.1% FAc) **Flow rate:** 600 μL/min **Ion spray voltage:** −4500 V **RT(DH-CIT):** 11.31 min **Transitions: DH-CIT:** 267/203/231	DH-CIT: 0.1	DH-CIT: 0.1	[40]
**Urine**	5 mL	Dilution with 5 mL of 1 mM acetic acid in water, mixed for 15 min.	---	**IAC CitriTest:** Wash: twice with 5mL distilled water. Elution: 4 mL of MeOH. Evaporation at 40 °C. Re-dissolution in 500 μL MeOH. Filtration through a Teflon syringe filter (0.45 μm)	**LC-MS/MS** Quadrupole MS/MS ESI negative mode MRM	**Column:** Nucleosil^®^ 100-5 C18 HD 125 × 3 mm (21 °C). **Mobile phase:** **A:** ammonium formate 1 mM in water; **B:** ammonium formate 1 mM in MeOH **Flow rate:** 0.2 mL/min **RT(CIT):** 9.3 min **RT(DH-CIT):** 8.7 min **Transitions:** **CIT:** 249.0→204.7 *m*/*z*→176.7 *m*/*z* (CE (eV): 15 and 19) **DH-CIT:** 265.0→221.0→176.7 *m*/*z* (CE (eV): 16 and 20)	CIT:0.02 DH-CIT: 0.05	CIT:0.05 DH-CIT: 0.10	[5,18]
**Urine**	500 µL	Centrifugation (5600× *g*, 3 min). Incubation with 500 µL PBS (pH 7.4) containing β-glucuronidase for 16 h at 37 °C.	---	**Oasis PRiME HLB:** Wash: 2 × 500 µL H_2_O. Elution: 3 × 200 µL ACN. Evaporation. Re-dissolution: 470 µL mobile phase.	**UHPLC-MS/MS** ESI positive mode MRM.	**Column:** Acquity HSS T3 column (2.1 × 100 mm) with 1.8 µm, at 35 °C. **Mobile phase:** **A:** water; **B:** ACN (both acidified with 0.1% HAc) **Injection volume:** 10 µL **Ion spray voltage:** −4500 V **Transitions:** **DH-CIT:** 265.0→221.1→246.9 **CIT:** 251→233.2→205.2 **RT(DH-CIT):** 15.9 min **RT(CIT):** 18.9 min	CIT: 0.003 DH-CIT: 0.003	CIT: 0.01 DH-CIT: 0.01	[42]
**Urine**	2 mL	Mix with 18 mL of ACN/H_2_O/FAc (52/45/3, *v*/*v*/*v*) in a 50 mL centrifuge tube.	**QuEChERS:** Mixture of 4 g of MgSO_4_ and 1 g of NaCl in the extraction tube. Vigorously shaking. Centrifugation (4000× *g*, 6 min), 5 mL of the organic layer were evaporated to dryness. Re-dissolution in 0.5 mL of H_2_O/MeOH (85/15, *v*/*v*). Filtration with PVDF (0.22 μm)	---	**UPLC-MS/MS** (TQS mass spectrometer) ESI negative mode.	**Column:** HSS T-3 column (2.1 × 100 mm, 1.8 μm). **Mobile phase:** **A:** Water/MeOH/HAc (94/5/1, *v*/*v*/*v*) **B:** MeOH/water/HAc (97/2/1, *v*/*v*/*v*), both buffered with 5mM ammonium acetate **Flow rate:** 0.3 mL/min **Source and desolvation temperature:** 200 °C; **Desolvation gas flow:** 550 L/h. **RT(CIT):** 7.02 min **Transitions:** 281→205→249;	CIT: 0.5	CIT: 1.0	[38]
**Urine**	2 mL	Mix with 18 mL of ACN/H_2_O/FAc (53/44/3, *v*/*v*/*v*) in a 50 mL centrifuge tube.	**QuEChERS:** 4 ± 0.05 g of Mg SO_4_ and 1 ± 0.01 g of NaCl were added into the extraction tube. Shaking (30 min) Centrifugation (4000× *g*, 6 min). Evaporation of 5 mL of the supernatant to dryness (40 °C). Re-dissolution in 500 μL of H_2_O/MeOH (90/10, *v*/*v*). Centrifugation (10,000× *g*, 6 min). Filtration.	---	**UPLC-MS/MS**	Chromatographic conditions identical to those used by Martins et al. [38].	CIT:0.14	CIT:0.20	[15]
**Plasma**	1 mL	Mix with 1 mL ACN (1:1, *v*/*v*) to precipitate protein.	Centrifugation (9300 rpm, 3 min). Evaporation of 1 mL of the upper layer (40 °C). Reconstitution in 300 µL MeOH.	---	**HPLC-FD:** λ exc.: 330 nm λ em.: 500 nm **LC–MS/MS**	Chromatographic conditions identical to those used for urine by Blaszkewicz et al. [22].	CIT: 0.07	CIT: 0.15	[22]
**Plasma**	1 mL	Mix with 1 mL ACN (1:1, *v*/*v*) to precipitate protein.	Centrifugation (12,000 rpm, 3 min). Evaporation of 1 mL of the upper layer (40 °C) Reconstitution in 350 μL of MeOH Centrifugation (12,000 rpm, 3 min) Filtration through a Teflon syringe filter (0.45 μm).	---	**LC-MS/MS**	Chromatographic conditions identical to those used for urine by Ali et al. [5,18].	CIT: 0.07 DH-CIT: 0.15	CIT: 0.15 DH-CIT: 0.30	[43]
**Plasma**	1 mL	Mix with 1 mL ACN (1:1, *v*/*v*) to precipitate protein.	Centrifugation (9800× *g*, 3 min). 1 mL of the upper layer was evaporated to dryness (40 °C). Reconstitution in 350 μL MeOH. Centrifugation (9800× *g*, 3 min). Filtration through a Teflon syringe filter (0.45 μm).	---	**LC-MS/MS**	**Column:** Nucleosil^®^ 100-5 C18 HD (125 × 3 mm). **Mobile phase:** **A:** H_2_O containing 1 mmol/L ammonium formate **B:** MeOH containing 1 mmol/L ammonium formate. **RT(CIT):** 9.3 min **RT(DH-CIT):** 8.7 min Chromatographic conditions identical to those used by Blaskewicz et al. [22] and Ali et al. [43].	CIT: 0.07 DH-CIT: 0.15	CIT: 0.15 DH-CIT: 0.30	[36]
**Plasma**	1 mL	Mix with ACN/H_2_O/FAc (53/44/3, *v*/*v*/*v*).	Extraction conditions identical to those used by Martins et al. [37].	Clean-up conditions identical to those used by Martins et al. [38].	**UPLC-MS/MS**	Chromatographic conditions identical to those used by Martins et al. [37]. **Transitions:** 281.0→249.0→205.0 *m*/*z* **RT(CIT):** 8.5 min	CIT: 0.04	CIT: 0.09	[15]
**Plasma**	200 μL	Add 50 μL β-glucuronidase. Incubate overnight at 37 ± 2 °C in a water bath.	Then 1mL of ACN/HAc (99/1, *v*/*v*) were added to plasma samples and vortexed (30 s). *dilute and shoot* (DaS)	Centrifugation (5000 rpm, 10min). Evaporation of supernatant using gentle N_2_ stream (45 ± 5°C). The dry residue was reconstituted in 200 μL of ACN/H_2_O (10/90, *v*/*v*). Mix in vortex (30 s). Filtration through a Nalgene syringe filter (0.22 μm). A 10μL aliquot was injected.	LC-MS/MS ESI positive mode	**Column:** 2.6μm Kinetex 100 RP-18 (100 mm × 2.1mm i.d.) (40 °C). **Mobile phase:** **A:** 0.2 mmol/L aqueous HAc sol.; **B:** ACN **RT(CIT):** 5 min **Transitions:** 251.1→233 *m*/*z* (CE (V): 24)	CIT: 0.18	CIT: 0.44	[49]

ACN—acetonitrile; ACT—acetone; DCM—dichloromethane; DMSO—Dimethyl Sulfoxide; ES—extraction solvent; ESI—electrospray ionization; FAc—formic acid; HAc—glacial acetic acid; HCl—hydrochloric acid; Hex.—hexane; H_2_O—water; *g*—centrifuge force; IAC—immunoaffinity column; MeOH—methanol; MgSO_4_—magnesium sulphate; MRM—multiple reaction monitoring; N_2_—nitrogen; NaCl—sodium chloride; Na_2_CO_3_—sodium carbonate; PVDF—polyvinylidene fluoride; RC—regenerated cellulose membrane; RT—retention time; SPE—solid phase extraction; TFA—trifluoracetic acid; TSF—Teflon syringe filter.
